# Analysis of proteins with the 'hot dog' fold: Prediction of function and identification of catalytic residues of hypothetical proteins

**DOI:** 10.1186/1472-6807-9-37

**Published:** 2009-05-28

**Authors:** Lakshmi S Pidugu, Koustav Maity, Karthikeyan Ramaswamy, Namita Surolia, Kaza Suguna

**Affiliations:** 1 Molecular Biophysics Unit, Indian Institute of Science, Bangalore, 560 012, India; 2 Molecular Biology and Genetics Unit, Jawaharlal Nehru Centre for Advanced Scientific Research, Bangalore, 560 064, India

## Abstract

**Background:**

The hot dog fold has been found in more than sixty proteins since the first report of its existence about a decade ago. The fold appears to have a strong association with fatty acid biosynthesis, its regulation and metabolism, as the proteins with this fold are predominantly coenzyme A-binding enzymes with a variety of substrates located at their active sites.

**Results:**

We have analyzed the structural features and sequences of proteins having the hot dog fold. This study reveals that though the basic architecture of the fold is well conserved in these proteins, significant differences exist in their sequence, nature of substrate and oligomerization. Segments with certain conserved sequence motifs seem to play crucial structural and functional roles in various classes of these proteins.

**Conclusion:**

The analysis led to predictions regarding the functional classification and identification of possible catalytic residues of a number of hot dog fold-containing hypothetical proteins whose structures were determined in high throughput structural genomics projects.

## Background

**Figure 1:**
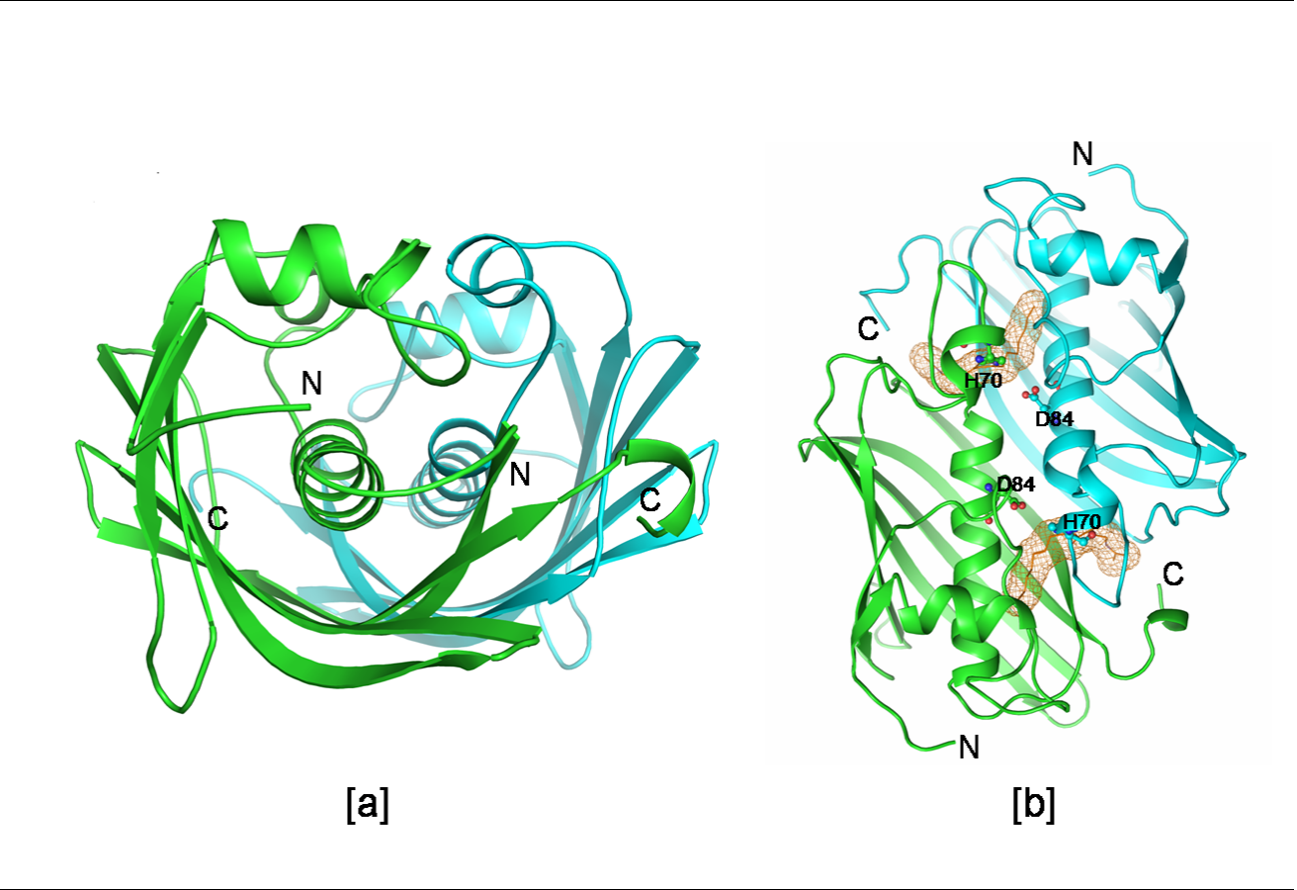
**Dimer of FabA from *E. coli***. Figure showing the a) hot dog fold b) active site tunnels (PDB code: 1MKA). The two views are related by a rotation of 90° about the horizontal axis passing through the centre of the molecule. Catalytic residues are shown in ball and stick representation. The inhibitor 3-decenoyl-NAC is shown in mesh representation for highlighting the tunnels. All the figures except Figure 9 are made in PyMOL [DeLano Scientific LLC].

The 'Hot dog' fold was first identified in the crystal structure of β-hydroxydecanoyl thiolester dehydratase (FabA) from *E. coli *[1]. The hot dog fold in this structure is formed by a sausage-like long central helix wrapped around by a highly curved six-stranded β-sheet resembling the bun (Fig. 1). Several protein structures determined since then were shown to contain this fold. These proteins take part in several functions including the dehydration step of fatty acid elongation, thioester hydrolysis, transcription regulation in fatty acid metabolism and degradation of phenylacetic acid and environmental pollutants.

It appears that the hot dog fold is designed for coenzyme A (CoA) binding in cellular processes involving fatty acids and related molecules. The binding site in FabA has been identified as a well-formed deep tunnel present at the subunit interface [1]. Residues from both subunits contribute to the formation of the tunnel which is hydrophobic in nature except at the two catalytic residues His70 and Asp84. FabA, being a homodimer, consists of two tunnels related by a twofold symmetry. The active site is located slightly below the surface of the protein and the active residues at each site come from a different subunit.

Dillon and Bateman [2] have unified the large superfamily of hot dog fold proteins by an extensive analysis of their sequences. They have classified a large number of proteins from prokaryotes, archaea and eukaryotes of this superfamily into 17 subfamilies based on the clustering of sequences and available structural and biochemical information. Consensus sequence motifs were identified by them for each subfamily. Though all these proteins possess the hot dog fold, the inter-subfamily sequence similarities are very low (10–20%). They showed that the hot dog fold domain, though exists as a single entity of the coded proteins, also associates with other domains, suggesting the occurrence of domain fusion events involving this domain. It has been found to be associated with a second hot dog fold domain in tandem and also with various types of other domains such as LpxC (UDP-3-O-acyl N-acetylglucosamine deacetylase), AMP binding, acyl transferase, aldehyde dehydrogenase and a few more [2].

In the last two years, there has been an enormous increase in reports of the structures of hot dog fold containing proteins and the deposition of their coordinates in the Protein Data Bank. Currently, the PDB contains more than sixty non-redundant crystal structures of proteins with the hot dog fold, a majority of them being hypothetical proteins taken up by structural genomics programs, for which no other characterization has been reported until now (see Additional file 1). The availability of a reasonable number of crystal structures of hot dog fold proteins facilitated a more detailed comparative analysis across the subfamilies. Here, we attempt to group these hot dog fold proteins into their corresponding subfamilies and analyze the structure-function relationships of the subfamilies having representative crystal structures. This paper describes a detailed structural comparison of the hot dog fold proteins and their different modes of quaternary associations and functional diversity. This analysis made it possible to predict functional subfamilies and identify possible catalytic residues for a number of hypothetical proteins with known structures which should aid in further experimental verifications.

## Results and Discussion

### Diversity in quaternary association and function

**Figure 2:**
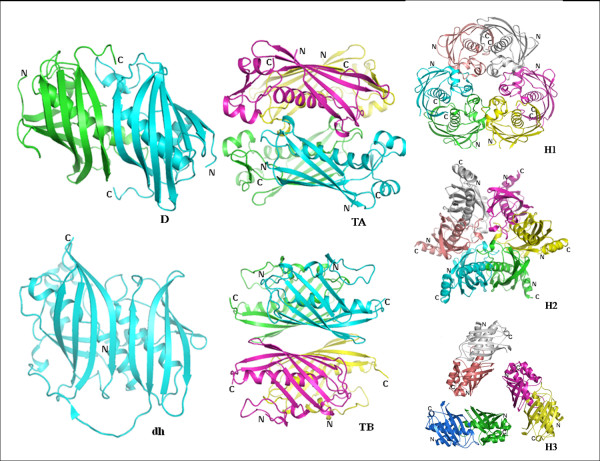
**Various quaternary associations of hot dog fold proteins**. D – dimer (PDB code: 1MKA); dh – double hot dog (PDB code: 1C8U); H1 – hexamer with active site loops at interface (PDB code: 1Z6B); H2 – hexamer with N-terminal helices at interface (PDB code: 1YLI); H3 – hexamer with head-to-tail arrangement of dimers (PDB code: 2PFC); TA – tetramer with central helix interactions (PDB code: 1BVQ); TB – tetramer with back to back stacking of the β-sheets (PDB code: 1Q4T). D_dh_A (dimer of double hot dog with central helix interactions) is similar to TA while D_dh_B (dimer of double hot dogs with back to back interaction of the β-sheets) is similar to TB. Tr_dh _(trimer of double hot dogs) is similar to H2.

Each subunit of a hot dog fold is made up of 5 or 6 highly curved antiparallel β-strands wrapped around a long α-helix. Our analysis indicated that the basic structural repeat unit of the hot dog fold proteins is either a homodimer of two such subunits or a double hot dog with a single polypeptide chain folding into two hot dog domains (Fig. 1). The dimer of hot dog folds is formed by the association of β-sheets from both the subunits to form a continuous anti-parallel β-sheet. The two helices run antiparallel to each other at the dimer interface. In the enzymes with the repeat unit as the dimer of hot dogs, the active sites comprise residues from both the subunits. In the case of double hot dogs, the two domains have an undetectable sequence similarity (~10% identity as derived from structure-based alignment). However, at the nucleotide level, the sequence identity between the two domains of the double hot dogs is around 50% [3]. Though these basic building blocks (homodimer or double hot dog) of various subfamilies are similar with root mean square deviations (rmsd) of 1.0–3.0 Å for the Cα atoms, they differ in their quaternary associations and are highly divergent in their sequences. The various quaternary associations observed are dimers (D); tetramers with central helix interactions at the tetramer interface (TA); tetramers with back to back stacking of the β-sheets of the repeating units (TB); hexamers with active site loops at the interface (H1); hexamers with N-terminal helices at the interface (H2); hexamers with head-to-tail arrangement of dimers (H3); dimers of double hot dogs with central helix interactions (D_dh_A); dimers of double hot dogs with back to back stacking of β-sheets (D_dh_B); and trimers of double hot dogs (Tr_dh_) which appear like H2 (Fig. 2). The two domains of the double hot dogs are connected by long loops. Though they connect the second β-strand from the centre in the first subunit to the last β-strand of the second subunit in all the cases, the loops have considerable variation in length and conformation and also in the direction they follow to connect the subunits. This variation in the nature of the connecting loops appears to be linked with the quaternary association (D_dh_A, D_dh_B and Tr_dh_) of the double hot dog fold proteins. The analysis of buried surface areas of various types of quaternary association has revealed that approximately 17–20%, 20–25%, 26–30%, 32–36%, 26–30% and 16% surface area per monomer gets buried upon the formation of D, TA, TB, H1, H2 and H3 types of quaternary associations, respectively.

An analysis of these proteins indicates the following general features: The hot dog fold domain does not exist as a single entity. It always forms a dimer (or a double hot dog) or higher oligomers. There are different modes of association of the basic building blocks in higher oligomers, but strict correlation between oligomeric state and function has not always been observed. Subfamilies with hot dog fold proteins of similar quaternary associations differ in their substrate specificities. On the other hand, proteins with different quaternary associations and highly divergent sequences can carryout the same reaction as in the case of 4-hydroxybenzoyl-CoA thioesterases. In all the crystal structures considered, the consensus sequence motifs identified earlier [2,4] are localized at the active site and at the interface of the quaternary association indicating that the information about the substrate specificity and quaternary association are encoded in the sequence. Variations of the consensus sequence at the interface change the oligomeric state. The double hot dog domains are found to accommodate bulkier substrates and generally contain one active site per dimer instead of two. The structure-function relationships of subfamilies with the available representative crystal structures (see Additional file 1 and 2) are discussed here.

### Subfamilies of the hot dog fold suprefamily of proteins

#### 1. Dehydratases (FabA and FabZ)

The two subfamilies of dehydratases, FabA-like dehydratases and FabZ-like dehydratases have been well studied both biochemically and structurally. Both FabA and FabZ are isozymes catalyzing the third step of elongation in Type II fatty acid biosynthesis. FabZ is only a dehydratase while FabA has an additional isomerase activity. The biological oligomer of FabZ is a hexamer [5-7], a trimer of dimers where the active site loops are stabilized by the interactions at the hexameric interface. However, FabA is active as a dimer [1]. Dimers of FabA and FabZ superpose with a rmsd of around 1.5 Å and the positions of the catalytic dyad (Glu-His in the case of FabZ and Asp-His in the case of FabA) are structurally conserved. The two *cis *peptides Tyr-Pro (FabZ)/Ala-Pro (FabA) in the N-terminal loop lining the active site tunnel and His-Phe harbouring the catalytic histidine are conserved in both the classes of enzymes. However, there is a subtle difference in the overall architecture of the active site tunnels, which was attributed to the additional isomerase activity of FabA [8]. It was shown by domain-swapping experiments that the β-strand at the dimer interface and the loop connecting this β-strand to the next one are important for isomerase activity [9]. In the case of FabA, consensus sequence motifs are localized to the loops that form the active site tunnel and the region responsible for the isomerase activity, while in the case of FabZ, the consensus sequence motifs are located on active site loops which are also involved in hexameric contacts. When a hexamer of FabA was generated by superimposing three dimers onto the three dimers of hexameric FabZ, severe steric clashes of less than 1 Å were observed at the dimer-dimer interfaces between the atoms of the loops containing the catalytic histidine and the loop involved in isomerase activity, thus indicating the neccessity of the dimeric state of FabA to function as a dehydratase/isomerase.

Each step of the FAS II pathway present in bacteria and plants is catalyzed by a distinct enzyme. In contrast to this, in the FAS I pathway of mammals and fungi, all the reactions are catalyzed by a large single multienzyme complex called the synthase. The dehydratase domain identified in each subunit of the homodimeric porcine fatty acid synthase structure (PDB code: 2CF2[10]) was found to have the double hot dog fold flanked by FabD (malonyl-CoA/acyl-CoA-ACP transacylase) and FabI (NADPH-dependent β-enoyl reductase) domains. As the structure is available at a low resolution of 4.5 Å and the deposited coordinates contain only the Cα atoms of the fitted model, it is not included in further analysis presented in the paper.

#### 2. Thioesterases

This group of enzymes catalyzes the hydrolysis of the thioester bond in fatty acids bound to CoA or acyl carrier protein (ACP) [11]. The thioesterases belong to different subfamilies of hot dog fold proteins as they differ in their substrate specificities and are also highly divergent in sequences. Eight of the seventeen subfamilies [2] of hot dog fold proteins are thioesterases and representative crystal structures are available for all of these thioesterase subfamilies (see Additional file 1).

#### a. Acyl-CoA thioesterases

**Figure 3:**
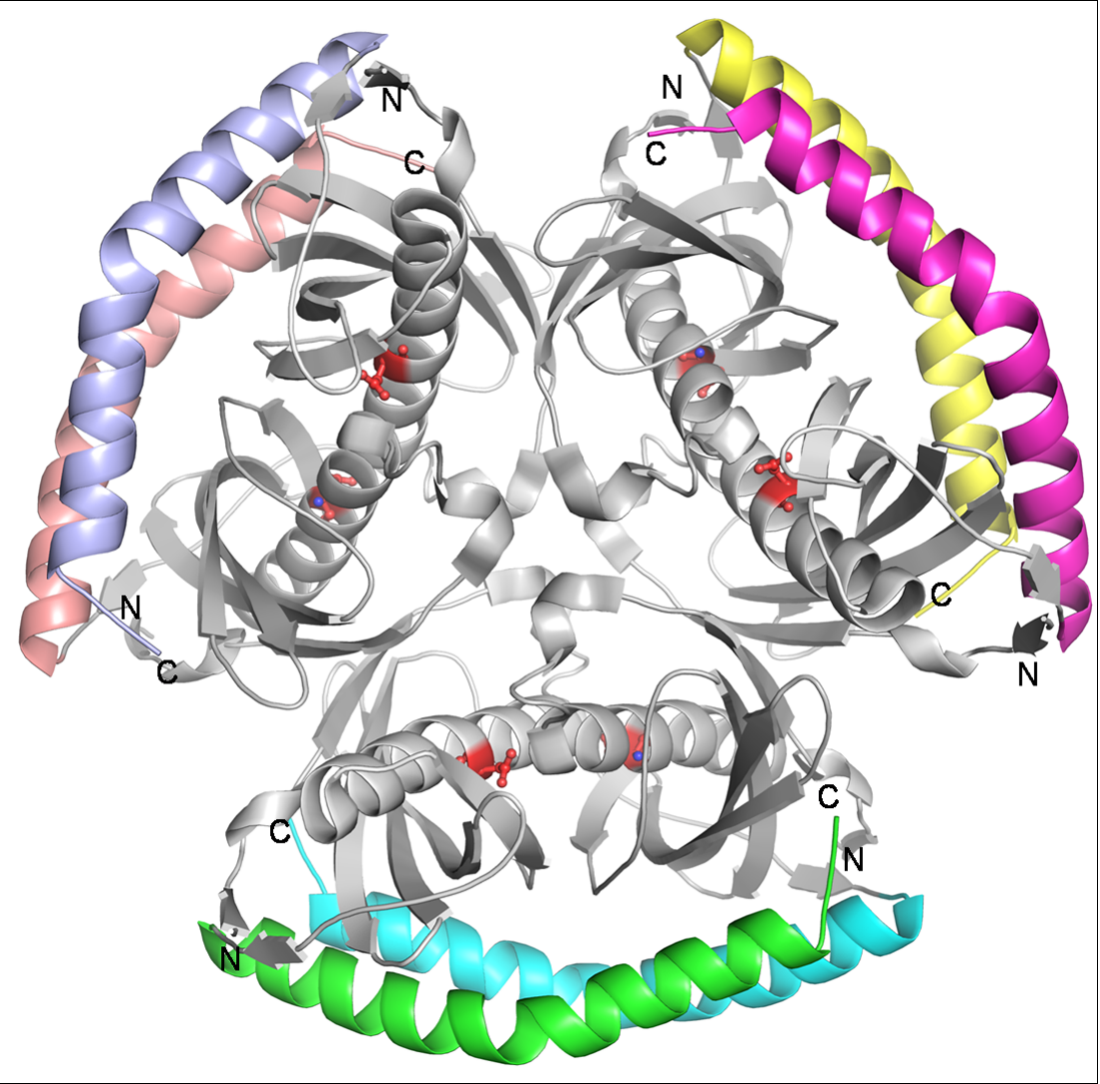
**Hexamer of *B. cereus *acyl-CoA thioesterase**. It shows the extra C-terminal helices that wrap around the H2 type of hexamer (PDB code: 1Y7U). Putative catalytic Asp residues are shown in red.

Acyl-CoA thioesterases are widespread in prokaryotes and are also found in a few eukaryotes. In mammalian systems, these enzymes have a double hot dog domain and a START (StAR related lipid-transfer) domain while in others the basic building block is the dimer of hot dogs. Crystal structures with PDB codes: 1VPM (*B. halodurans*), 1YLI (*H. influenzae*), 1Y7U (*B. cereus*), 2GVH (*A. tumefaciens*), 2QQ2 (Human acyl-CoA thioesterase 7), 2EIS (*T. thermophilus*) and 3B7K (human acyl-CoA thioesterase 12) from structural genomics programs have the consensus sequence of acyl-CoA thioesterases. All these structures show H2 type hexamer formation while 2GVH and 3B7K form trimers of double hot dog domains (Tr_dh_). Asp44 of the *H. influenzae *enzyme (1YLI) [12] has been identified as the catalytic residue by mutagenesis. Structures of the N- and C-terminal domains of the mouse Acyl-CoA thioesterase 7 were determined separately (PDB codes: 2V1O and 2Q2B respectively) and Asp213 along with Asn24 were found to take part in catalysis. Though the individual domains of this protein form H2 type of hexamers of the N-terminal domain and dimers of the C-terminal domain, the full length protein was shown to be a trimer with Tr_dh _arrangement in solution [13]. The catalytic aspartate residue found in the reported structures is conserved in the rest of the hypothetical proteins (see Additional file 2) and thus could be identified as the catalytic residue in these enzymes. Some of the proteins in this class possess an additional long helix at the C-terminus (Fig. 3). These helices of varying lengths in different structures wrap around the hexamer.

#### b. TesB-like thioesterases

TesB-like thioesterases found mostly in bacteria and eukaryotes include the human peroxisomal enzyme that binds to the HIV Nef protein. The crystal structure of TesB, the medium chain acyl-CoA thioesterase II from *E. coli *(EcThII; PDB code: 1C8U) for the first time showed the structure of a double hot dog. The biologically active oligomeric state is a dimer of double hot dogs (D_dh_B) with the consensus sequence at the dimer interface and the active site. The dimer is formed by the back to back stacking of the 12 stranded β-sheets. The crystal structure revealed a novel catalytic mechanism wherein a hydrogen-bonded triad of Asp204, Thr228 and Gln278 are identified by site directed mutagenesis as catalytic residues and synergistically activate a water molecule for nucleophilic attack [14]. The crystal structure of a putative peroxisomal thioesterase from yeast (PDB code: 1TBU) with the hot dog fold structure forms a tetramer of type TB and has only the first few consensus sequence motifs which do not include the catalytic triad.

#### c. YbgC-like thioesterases

The YbgC-like thioesterases, that belong to the *tol-pal *cluster, act on short chain aliphatic acyl-CoA as shown in the gamma-proteobacterium *Haemophilus influenzae *[15]. This *tol-pal *cluster operon is well conserved in all gram-negative bacteria and is shown to be crucial for the maintenance of cell envelope integrity [16]. The crystal structures of the hypothetical proteins with PDB codes: 1S5U (*E. coli*), 1Z54 (*T. thermophilus*), 2GF6 (*S. solfataricus*), 2HX5 (*P. marinus*) and 2EGJ (*A. aeolicus*) have a hot dog fold in the subunits and a consensus sequence of the YbgC-like subfamily. Further, a tetramer of the type TA is formed within the asymmetric unit or with the symmetry-related molecules, indicating that the tetramer might be the biologically active oligomer. Zhuang et al. (2002) [15] have shown in the case of the *H. influenzae *YbgC protein that the D18N mutant was not capable of hydrolyzing the short chain aliphatic acyl-CoA thioesters, thus suggesting a catalytic role for this aspartate residue. This residue present in the consensus sequence motif is conserved in all the proteins (see Additional file 2) mentioned above.

#### d. 3-hydroxyacyl-CoA dehydrogenase-associated thioesterases

3-hydroxyacyl-CoA dehydrogenase-associated thioesterases are specific to short chain fatty acids of fatty acid metabolism catalyzing the reduction of 3-hydroxyacyl-CoA to 3-oxoacyl-CoA [17]. The crystal structure of a hypothetical protein from *Pseudomonas putida *(PDB code: 2HLJ) possesses the conserved sequence motif of the hydroxyacyl-CoA dehydrogenase subfamily. One hot dog subunit is present in the asymmetric unit and forms a dimer with a symmetry-related molecule with the consensus sequence motif present at the active site.

#### e. 4-Hydroxybenzoyl-CoA thioesterases

4-Hydroxybenzoyl-CoA thioesterases, encoded by the *fcbC *gene, are involved in the degradation of the environmental pollutant 4-chlorobenzoate to 4-hydroxybenzoate. The 4-chlorobenzoyl-CoA degradation pathway operons of certain *Pseudomonas *and *Arthobacter *bacterial strains show significant differences in gene order and sequences [18]. Both the subfamilies (i) 4HBT-I (from *Pseudomonas *sp. strain CBS-3; PDB code: 1BVQ[19], 1LO7[20]) and (ii) 4HBT-II (from *Arthobacter *sp. strain SU*; PDB code: 1Q4T) have been studied both biochemically and structurally. Though both the enzymes form tetramers, they have different modes of quaternary association, CoA binding sites and catalytic architecture. In the case of 4HBT-I, TA tetramers are formed, while 4HBT-II enzymes form TB tetramers. The structures of these two enzymes in complex with inhibitors revealed that the 4-hydroxy phenacyl moieties are oriented such that the thioester C = O can form a hydrogen bond with the amide nitrogen atoms of Tyr24 and Gly65 located at the N-terminus of the central helix in *Pseudomonas *(4HBT-I) and *Arthobacter *(4HBT-II), respectively. Due to the hydrogen bond and the polarity of the helix, the C = O group is polarized, making it susceptible to nucleophilic attack. Though the catalytic mechanism is similar, the positions of the carboxylate residues, Asp17 in 4HBT-I (1BVQ) and Glu73 in 4HBT-II (1Q4T[21]), involved in catalysis are different. These two enzymes having the same fold and catalyzing the same reaction differ remarkably in their quaternary associations. In 4HBT-I the active site is formed at the dimer interface, while in 4HBT II three subunits contribute to the active site. A hypothetical protein (PDB code: 2OAF) has a consensus sequence motif of that of 4HBT-I and forms a TA tetramer. For 4HBT-II, the extra N-terminal motif, which is also one of the consensus motifs, is positioned before the central helix and prevents the formation of helix-to-helix packing observed in the quaternary association of 4HBT-I. The crystal structures of hypothetical proteins with PDB codes: 1O0I (*H. influenzae*), 1VH5 (*E. coli*) and 1VH9 (*E. coli*) have the consensus sequence of the 4HBT-II subfamily and a similar mode of quaternary association. In all these proteins, the catalytic machinery is both sequentially and structurally conserved within the subfamilies.

#### f. PaaI-Thioesterases specific to phenylacetic acid

**Figure 4:**
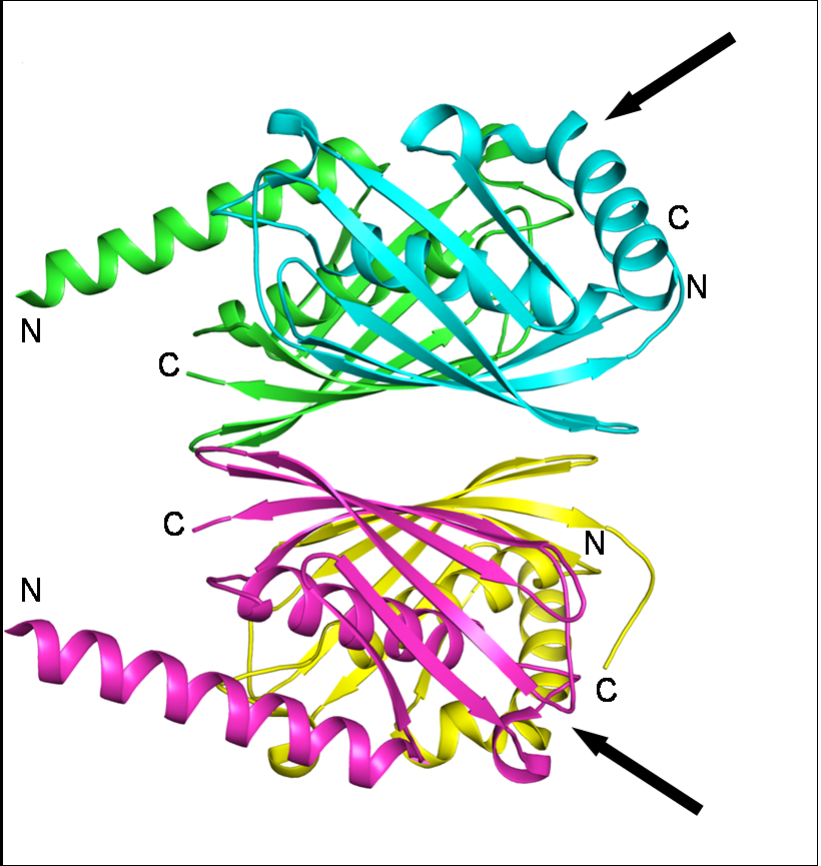
**TB tetramer of *E. coli *PaaI**. The kinked conformation of the N-terminal helices is indicated by arrows (PDB code: 2FS2).

**Figure 5:**
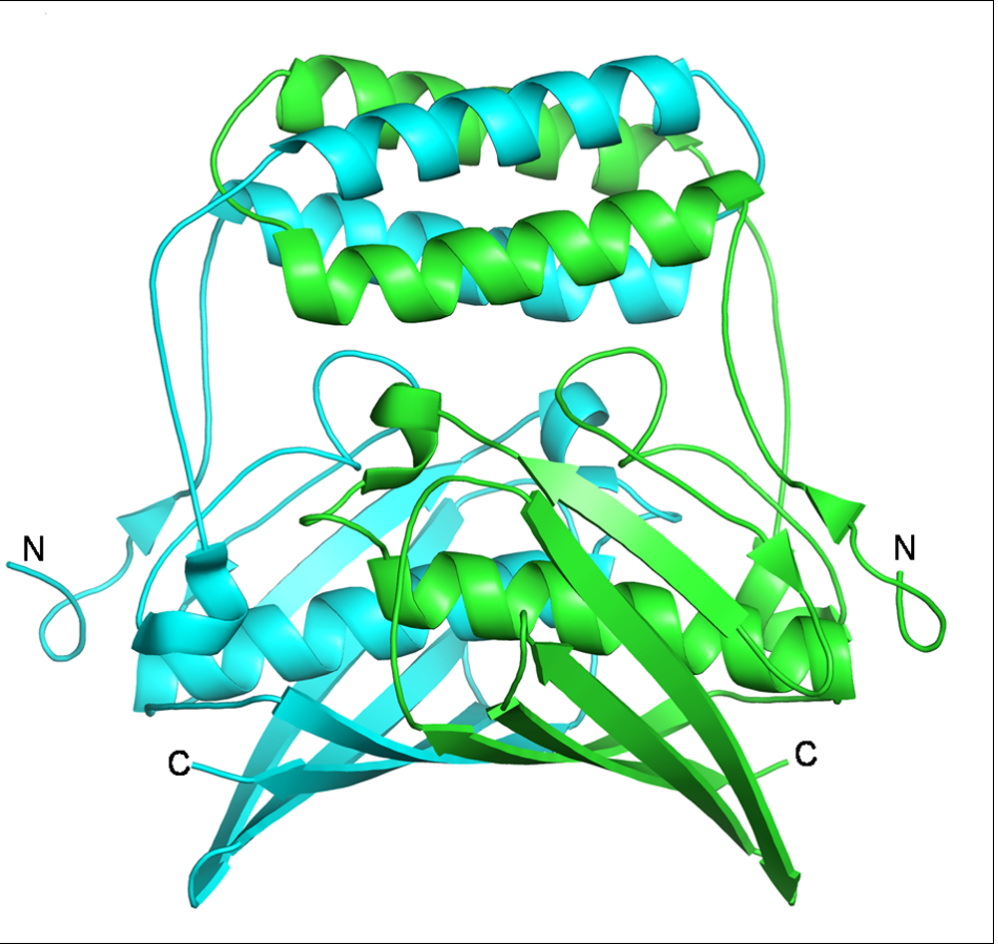
**Structure of PaaI from *Rhodococcus sp***. It has an additional four helix bundle as a separate domain (PDB code: 2OV9).

PaaI is a thioesterase involved in the phenylacetic acid catabolic pathway, catalyzing the degradation of phenylacetyl-CoA [22]. PaaI from *Thermus thermophilus *(TtPaaI; PDB code 1J1Y) and *E. coli *(EcPaaI: PDB code: 2FS2) have been characterized both structurally and biochemically. They also form TB tetramers similar to the 4HBT-II subfamily. A novel induced fit mechanism was observed in the case of TtPaaI, where only two of the four active sites accommodate the ligand, indicating half of the sites reactivity. A rotation of up to 2° of the subunits upon ligand binding introduces a negative cooperativity preventing the binding of ligands at the other two active sites [23]. In the case of EcPaaI, the overall structure is similar to that of TtPaaI except for the presence of an additional long N-terminal helix that lines the active site (Fig. 4).The helix has a kink in two subunits of the tetramer [24] giving rise to two different catalytic platforms. It was further confirmed by site directed mutagenesis of the active site residues in both conformations of the helix that the subunit with the kinked N-terminal helix is catalytically active [24]. Four hypothetical proteins (PDB codes: 1ZKI from *P. aeruginosa*, 2CY9 from *M. musculus*, 2QWZ from *Silicibacter sp.*, 2PIM from *R. eutropha*) and a putative human enzyme (2F0X[25]) form tetramers of the type TB similar to that of TtPaaI whereas another protein (3BBJ) is a double hot dog with a D_dh_B type of quaternary structure. Hypothetical proteins 1IXL (*P. horikoshii*) [26], 2HBO (*C. crescentus*), 2PRX (*S. loihica*) and 2OV9 (*Rhodococcus sp.*) possess the active site consensus sequence motif (see Additional file 2) of PaaI but show variation in the consensus sequence motif located at the tetramer interface (see Additional file 2). These four proteins form dimers of hot dogs. In TtPaaI, Asp48 identified as the catalytic residue [23], occurs in the consensus sequence motif and is found to be conserved in all the proteins of this subfamily. The variation in the second consensus sequence motif that is present at the tetramer interfaces, might be responsible for the proteins of this subfamily having different oligomeric structures. However, the substrate specificities of these proteins are yet to be confirmed. The structure of 2OV9 is different from the others with an additional long loop consisting of two helices from each subunit forming a four-helix bundle as a separate domain (Fig. 5).

#### g. Fat-Thioesterases specific to acyl-ACPs

Acyl-ACP thioesterases terminate the Type II fatty acid biosynthesis by hydrolysing the thioester bond between an acyl moiety and ACP [27]. Two gene classes, *fatA *and *fatB*, exist in higher plants, and differ in sequence and substrate specificities [27,28]. The Fat subfamily of hot dog fold proteins contains members from both FatA and FatB classes [2]. The crystal structures of hypothetical proteins from *B. thetaiotaomicron *(PDB code: 2ESS) and *L. plantarum *(PDB code: 2OWN) possess tetramers formed by the dimers of double hot dogs with the central helices pointing inward (D_dh_A). This central helix contains the consensus sequence of the Fat subfamily. These proteins share a sequence identity of >20% with FatA and FatB of *Arabidopsi*s *thaliana*. Therefore, these two hypothetical proteins can be considered as members of the Fat subfamily.

#### 3. (R)-specific enoyl-CoA hydratases or MaoC dehydratase-like subfamily

**Figure 6:**
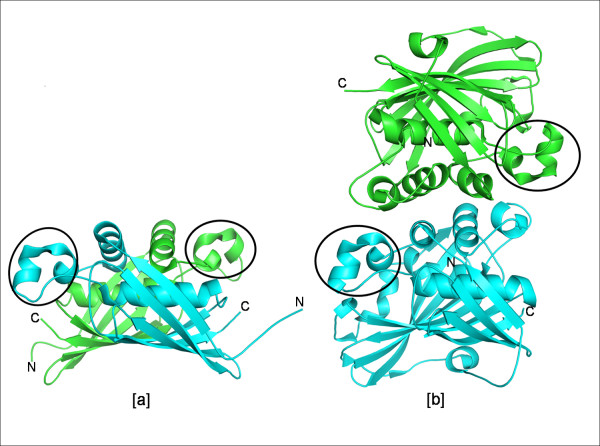
**(R)-specific enoyl hydratases**. a) prokaryotic (PDB code: 1IQ6) and b) eukaryotic (PDB code: 1PN2) origin. The structural motif of this subfamily – the overhanging segment is highlighted.

(R)-specific enoyl hydratase that catalyzes the hydration of *trans*-2-enoyl-CoA to (R)-3-hydroxyacyl-CoA is involved in supplying the (R)-3-hydroxyacyl-CoA from the fatty acid oxidation pathway to the polyhydroxy alkanoate (PHA) biosynthesis pathway [29]. The eukaryotic enzyme is found to be an integral part of a peroxisomal multifunctional protein (MFE-2 in fungi and MFE-1 in mammals) [4,30-32]. The crystal structures of a prokaryotic (R)-hydratase (PDB code: 1IQ6) from *Aeromonas caviae*, a eukaryotic hydratase 2 (PDB code: 1PN2) from *Candida tropicalis *and a human enzyme (PDB code: 1S9C) are available. In these structures, the loop corresponding to the one containing the catalytic histidine of the dehydratase FabA of *E. coli*, is longer by about 35 residues. This additional segment called the 'overhanging segment' contains both the catalytic residues Asp31 and His36 in 1IQ6. The prokaryotic enzyme forms a dimer of hot dogs with two such segments i.e., two catalytic sites, while the eukaryotic and human hydratases are dimers of double hot dogs, with each subunit having one catalytic site (Fig. 6). The double hot dog of eukaryotic and human hydratase 2, supposed to have arisen due to gene duplication, has structurally diverged during evolution to accommodate bulky fatty enoyl-CoAs at the cost of one catalytic site [3,33,34].

The consensus sequence motif for this subfamily has been identified by Qin et al. (2000) [4]. The current analysis suggests that the overhanging segment containing this sequence motif can also be considered as a structural motif of this subfamily as it is present in all the three proteins (1IQ6, 1PN2 and 1S9C) for which crystal structures and biochemical characterization are available. Three hypothetical proteins, one from *Mycobacterium tuberculosis *(PDB code: 2BI0[35]) and two from *Archaeoglobus fulgidus *(PDB codes: 1Q6W and 2B3M) can be classified into this subfamily. The first one is a trimer of double hot dog folds, the second one is a trimer of dimers and the third one is a dimer of hot dog folds indicating that the quaternary association is not conserved in this subfamily. Though all these proteins have hydratase activity, they differ in their substrate specificities. For example, recently cloned and characterized R-hydratases from the *P. aureginosa *PHA biosynthesis pathway, PhaJ1-PhaJ4 show a variation in activity based on the length of the fatty acyl chain of enoyl-CoAs [36]. PhaJ1 can act only on short chain enoyl-CoAs (C4–C6), while the other three can act on longer (C8–C12) enoyl-CoAs. The consensus sequence motif of the hydratase 2 domain [4] though present in this subclass of enzymes shows variation (see Additional file 2), reflecting the differences in quaternary association and substrate specificities. However, all the proteins of this subfamily share a sequence identity of around 15–40% and the catalytic dyad Asp31 & His36 is highly conserved both sequentially and structurally.

The crystal structures of FAS I synthase of yeast (PDB code: 2UV8[37]) and *Thermomyces lanuginosus *(PDB codes: 2UV9 and 2UVA[38]) are available at 3.1 Ǻ resolution. In contrast to the mammalian homodimeric enzymes, these two exist as α_6_β_6 _dodecamers. The dehydratase domain located on the β subunit has a unique triple hot dog fold. The first and the third hot dog domains associate as pseudodimers and are structurally similar to eukaryotic hydrates 2 enzymes having the double hot dog fold with the catalytic residues in the overhanging segment of the C-terminal domain. The second hot dog domain is inserted in the long loop connecting the first and the third hot dog domains in the same way the domains in the double hot dog structures are connected. The central helix of this domain is much shorter and has a different orientation compared to that in the typical hot dog domain described here. The yeast enzyme, which is well refined, has been included in the present analysis.

**Figure 7:**
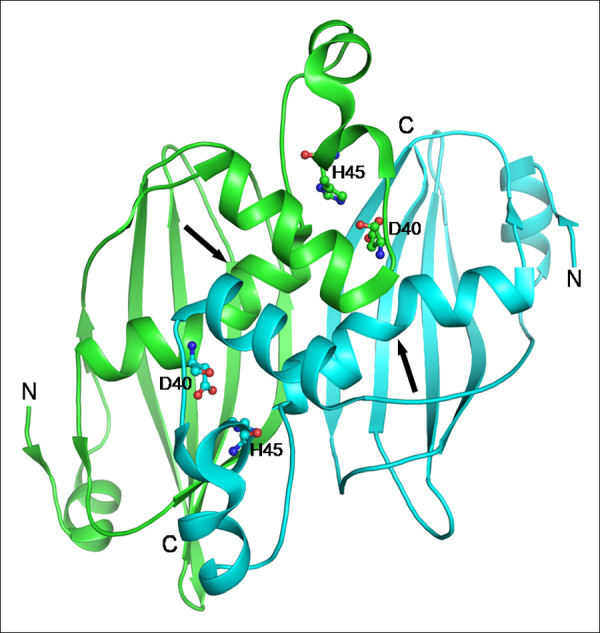
**Dimer of NodN-like hydratase from *M. tuberculosis***. The kink in the central α-helix shown by arrows (PDB code: 2C2I). Catalytic residues are shown in ball and stick representation.

The crystal structure of a hypothetical protein from *M. tuberculosis *(PDB code: 2C2I) possesses the consensus sequence motif of the NodN-like subfamily, a subgroup of MaoC like hydratases [39]. This protein is a dimer with two overhanging segments which contain the catalytic residues Asp40 and His45. The N-terminus of each subunit has an additional short β-strand and a small α-helix. A proline residue Pro71 at the centre of the central helix causes a 25° kink in the long helix (Fig. 7), giving rise to a curved long tunnel similar to eukaryotic and human hydratase 2 enzymes which are specific to longer substrates.

#### 4. YbaW subfamily

Structural genomics programs have shown that the crystal structures of conserved hypothetical protein YbaW (PDB codes: 1NJK from *E. coli*, 2ALI from *P. aeruginosa*, 2AV9 from *P. aeruginosa*, 2CYE from *T. thermophilus*, 2FUJ from *X. campestris *[40], 2NUJ from *Jannaschia sp. CCS1*, 2OIW from *B. stearothermophilus*) with the hot dog fold form tetramers of type TA similar to that of 4HBT-I. However, the sequences of both the subfamilies are quite different. Asp20 has been identified as the catalytic residue in the case of YbaW from *X. campestris *(PDB code: 2FUJ) which is conserved among all the other hypothetical proteins of this subfamily. This residue occupies the same position as His133 of *Plasmodium falciparum *FabZ enzyme (PDB code: 1Z6B). We have also seen a Glu/Asp residue in the consensus sequence (see Additional file 2) similar to Glu147 of PfFabZ.

#### 5. FapR subfamily

**Figure 8:**
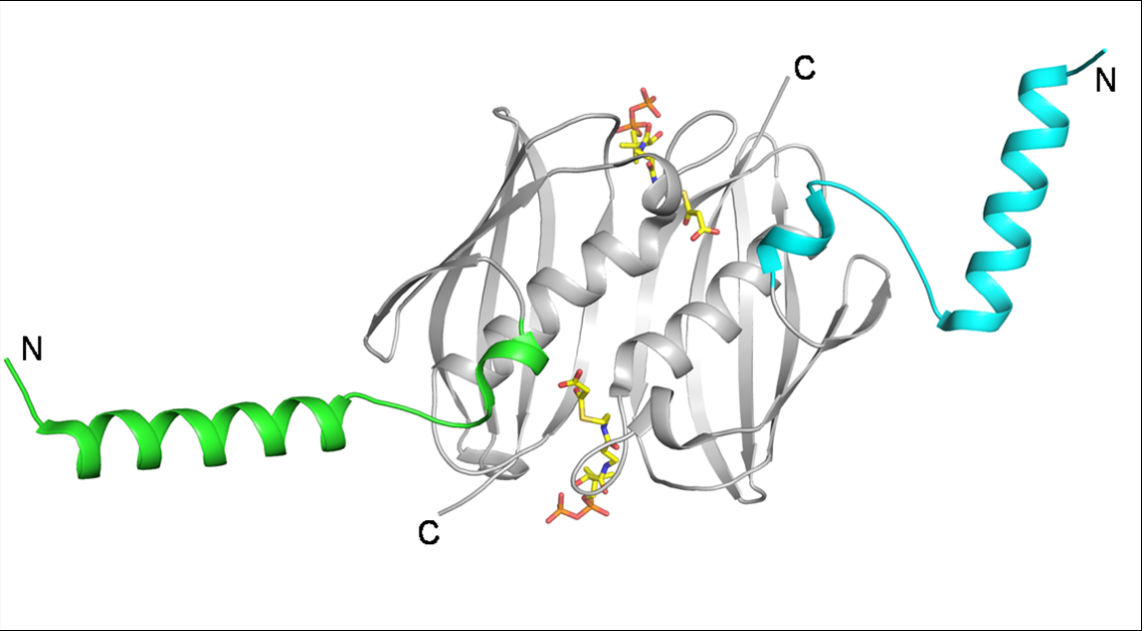
**Dimer of FapR from *B. subtilis***. The helix-turn-helix motif is highlighted and the bound substrate malonyl-CoA is shown in stick representation. (PDB code: 2F3X)

FapR is a transcriptional repressor of gene expression involved in Type II fatty acid biosynthesis and phospholipid biosynthesis in many gram-positive organisms [41]. It is highly conserved and acts as a negative regulator by binding to a consensus promoter sequence of *fap *regulon. Malonyl-CoA, an intermediate in the lipid biosynthetic pathway, regulates FapR. FapR contains a hot dog domain to accommodate malonyl-CoA and an additional helix-turn-helix motif at the N-terminus for DNA binding [2]. Recently reported crystal structures (Fig. 8) of the effector binding domain of the FapR from *B. subtilis *(PDB code: 2F3X[41]) have revealed that binding of malonyl-CoA induces significant structural changes in the three loops at the active site tunnel converting the open ligand binding groove into a long tunnel where the effector molecule binds. These conformational changes induced by ligand binding constrain the flexibility of a linker helix that connects the helix-turn-helix motif to the core and thus regulate transcription. A majority of hot dog fold proteins have catalytic functions, with rare exceptions such as FapR which has lost enzymatic action but plays a regulatory role.

#### 6. Acetyltransferases

The crystal structures of three hypothetical proteins (PDB codes: 1T82 from *S. oneidensis*, 1SH8 from *P. aeruginosa*, and 1YOC from *P. aeruginosa*) were found to be homodimers. These proteins share 27%, 38% and 34% sequence identity, respectively with the putative acetyltransferase YiiD from *E. coli*. However, the consensus sequence motif is not well conserved in these proteins. A glutamate residue in 1SH8 and 1YOC (see Additional file 2) has been identified as the catalytic residue by structural superpositions. However, the corresponding one is a threonine in 1T82.

### Other proteins with the hot dog fold

Other three subfamilies namely, CBS-associated, MSCP and AMP binding subfamilies [2] do not have representative crystal structures. On the other hand, two hypothetical proteins from *T. thermophilus *(PDB code: 2CWZ) and *T. maritime *(PDB code: 2Q78) possess the hot dog fold, but it was not possible to assign a subfamily to these proteins as they do not have any of the consensus sequence motifs of the known subfamilies. These proteins form a dimer of hot dogs. In addition, they have a two stranded β-sheet towards the N-terminus and a long helix towards the C-terminus. Phylogenetic analysis also shows that these proteins group together but do not cluster with any of the subfamilies. This might be a representative member of an unexplored subfamily of hot dog fold proteins. The yeast protein, a member of Phenazine biosynthesis, PhzF (PDB code: 1YM5[42]) has a kinked double hot dog with a similar domain structure but a domain association different from that described so far. Each hot dog subunit contains nine β-strands around the central α-helix which is shorter in length compared to those discussed so far.

The structure of Rv0098 from *M. tuberculosis *forms a new head-to-tail hexameric association (H3) made up of trimers of dimers of hot dog fold domains (PDB code: 2PFC[43]). This enzyme has been identified as a long chain acyl-CoA thioesterase with a catalytic site different from the known thioesterases with three residues Tyr33, Tyr66 and Asn74 stabilizing the transition state of the substrate.

### Structure-based phylogenetic analysis

**Figure 9:**
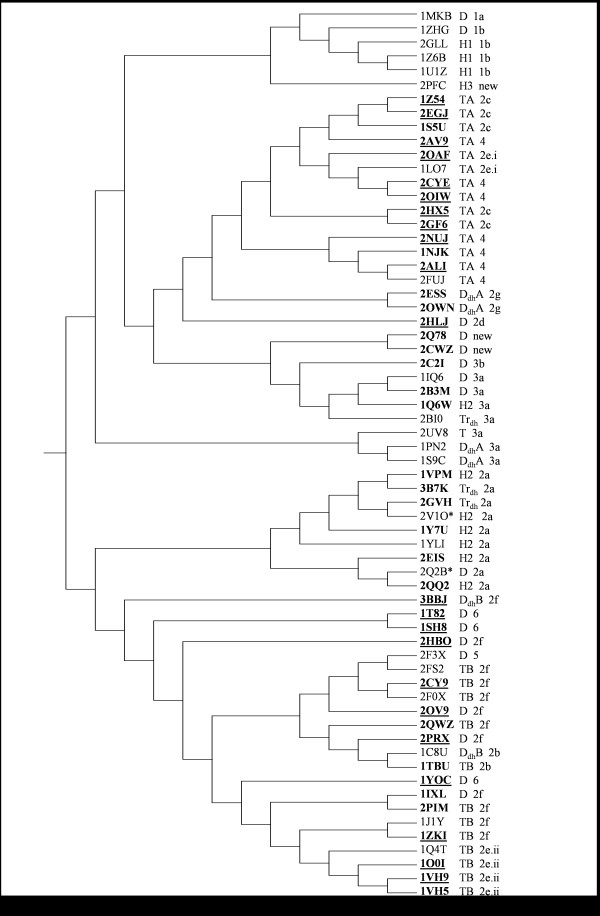
**Structure-based phylogenetic tree of hot dog fold proteins**. The corresponding quaternary association and the subfamily numbers (see Additional file 1 &2) are given next to the PDB code. *These two are the structures of the N- and the C-terminal domains of the double hot dog of mouse Acyl-CoA thioesterase 7, crystallized separately. The biologically relevant intact protein was shown to form Tr_dh _type of oligomer in solution [13].

Phylogenetic trees were generated for monomers and dimers with various options (neighbor-joining, minimum evolution, UPGMA) in the program MEGA [44]. Though both monomers and dimers yielded similar results, those with dimers were more consistent, one of which is shown in Figure 9. Though strict rules could not be drawn, certain distinct features appear in the phylogenetic trees. In some cases, all the proteins of a subfamily have the same quaternary structure: H1 in FabZ (1b), H2 in Acyl-CoA thioesterases (2a). All MaoC-like hydratases (Subfamily 3) cluster together with individual branches corresponding to different quaternary structures. All the TA type of tetramers group together though they belong to different subfamilies: YbgC (2c), YbaW (4) and 4HBT-I (2e.i). The related structures D_dh_A are also in close proximity. The TB type of tetramers are observed in subfamilies TesB-like thioesterase (2b), 4HBT-II (2e.ii) and PaaI (2f) which occur on neigbouring branches of the phylogenetic tree but are interspersed with a few dimers. This phylogenetic analysis further validates the functional annotation of the hypothetical proteins as they group into the respective subfamilies.

### CoA binding and catalytic sites

**Figure 10:**
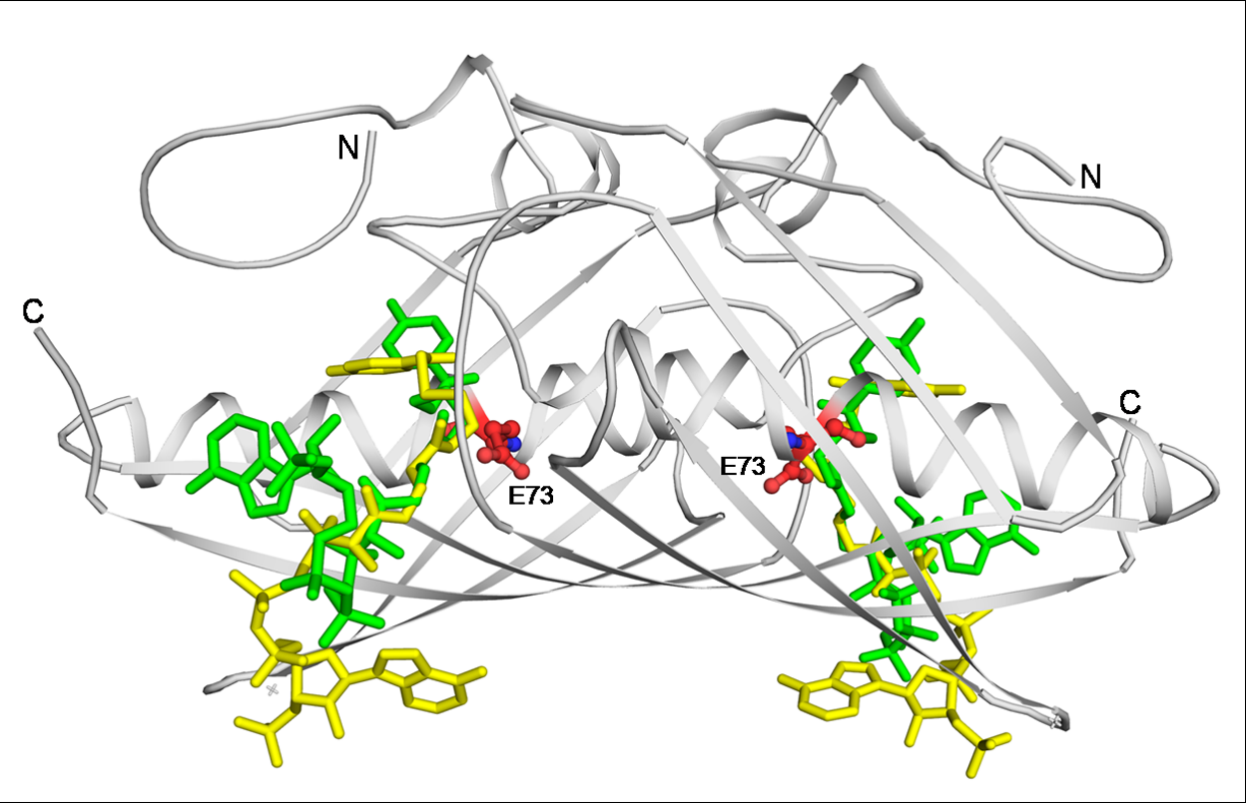
**Different modes of CoA binding**. The dimer of a 4HBT-II protein (PDB code: 1Q4T) is shown in cartoon representation and the bound CoA molecules in green. The CoA molecules of a 4HBT-I protein (1LO7) with a different binding mode are in yellow. Catalytic residues are shown in ball and stick representation.

Various substituents of acyl-CoA serve as substrates for most of the hot dog fold binding proteins. There are 12 crystal structures of various hot dog fold proteins complexed with different CoA molecules. The CoA molecule binds to the hot dog fold proteins in two different binding modes. In both cases, CoA binds near the active site in a curved conformation with the groups substituted at the 4'-phosphopantetheine arm located in the active site tunnel (Fig. 10). The β-mercaptoethylamine and pantothenate units are mostly solvent exposed and are stabilized by hydrogen bonds to the backbone of the C-terminal loop of the β-strand at the dimer interface. The major difference in the two modes of binding is the orientation of the nucleotide moiety. In the case of hot dog fold proteins with quaternary associations of the type D, TA, H2, D_dh_A, the 3'-phosphate interacts with the loop mentioned above and the nucleotide interacts with the convex surface of the curved β-sheet. In the case of hotdog fold proteins that have a quaternary association of the type TB and D_dh_B due to back to back stacking of the β-sheet, the 4'-phosphopantetheine arm winds in the opposite direction directing the nucleotide away from the β-sheet and is more solvent exposed. Within the TB mode of quaternary association, the 4HBT-II subfamily proteins show all site reactivity, while in PaaI from *T. thermophilus *half the site reactivity has been reported [23].

A structural comparison of the catalytic residues of various enzymes with those (Glu147 and His133) of FabZ of *P. falciparum *(PDB code: 1Z6B) revealed that while the catalytic sites are located in the same region, there is considerable variation in their architecture and catalytic residues as shown in Table 1. All H2-type hexamers of subfamily 2a have only one catalytic residue, Asp, similar to Glu147. One catalytic residue (Asp/Glu) similar to Glu147 of FabZ is present in all the enzymes which form TB-type tetramers, with additional Thr and Gln in one of the families. In TA-type tetramers, an Asp residue occupies the same position as His133 of FabZ. In subfamily 3, which shows a variety of oligomeric structures, the His residue is conserved but the position of the second catalytic Asp residue is slightly different from that of Glu147 of FabZ. Thus it appears that different types of active site architecture exist for different classes of hot dog fold proteins (subfamily 1, subfamily 2a, subfamily 3, all TA and all TB). These classes cluster together in the phylogenetic tree as well.

**Table 1:** Comparison of active site residues

**Subfamily**	**Biological assembly**	**Catalytic residues**		**Others**
1 a)	D	Asp	His	
b)	H1	Glu147*	His133*	

2 a)	H2	Asp	-	
b)	TB	Asp	-	Additional Thr & Gln
c)	TA	-	Asp	
e) i	TA	-	Asp	
ii	TB	Glu	-	
f)	TB & D	Asp	-	

3	D, D_dh_A, Tr_dh_, H2	-	His	Asp in a slightly different location

4	TA	-	Asp	

*Catalytic residues corresponding to FabZ of *P. falciparum *(PDB code: 1Z6B).

### Highly divergent sequences lead to similar structures

The present analysis shows correlations between the oligomerization, function and sequence of proteins with the hot dog fold (see Additional file 1, 2 and Figure 9). At the subunit level, the proteins possess highly divergent sequences but adopt a similar fold. A detailed comparison of these proteins revealed conserved hydrophobic interactions between the residues from the central helix and the various parts of the curved β-sheet. Though the residues involved in these interactions are from different parts of the sequence in different proteins, the interactions are structurally conserved and hence form the driving force for the formation of the hot dog fold.

### Ligand and pH induced Structural Changes

Two of the 64 proteins discussed in this analysis show structural changes upon ligand binding. In the case of *T. thermophilus *PaaI (PDB code: 1J1Y) protein a small rotaton of 2° in one of the dimers of the TB tetramer results in negative co-operativity and an asymmetric induced fit mechanism inducing half of the sites reactivity. In the case of *B. subtilis *FapR (PDB code: 2F3X), upon ligand binding, three flexible loops become ordered, a substrate binding tunnel is formed leading to conformational changes in the helix-turn-helix motif resulting in the impairment of DNA binding.

**Figure 11:**
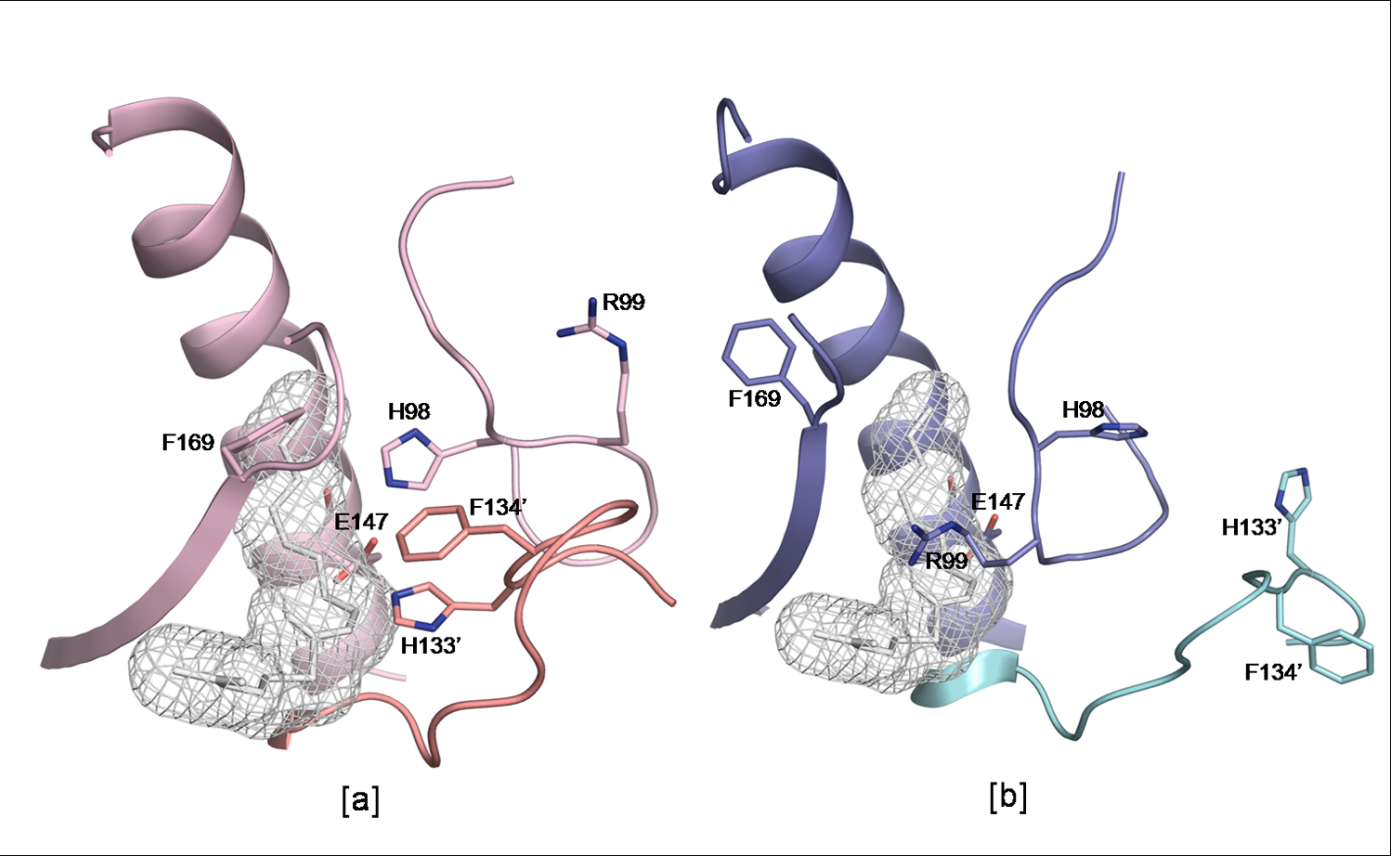
**The active site tunnel in different forms of *P. falciparum *FabZ**. a) active hexameric (PDB code: 1Z6B) and b) inactive dimeric form of FabZ from *P. falciparum *(PDB code:1ZHG). The inhibitor 3-decenoyl-NAC has been manually docked into the active sites and shown in mesh representation for highlighting the tunnels.

The third enzyme in which structural changes have been observed is FabZ of *P. falciparum *(PDB codes: 1Z6B &1ZHG). As reported earlier [6], the two *cis *peptide bonds in the active site loops of the active hexameric FabZ molecule flip to *trans *peptide bonds, leading to large conformational changes in the active site loops, by which the architecture of the catalytic site drastically changes making it incapable of binding to the substrate (Fig. 11). In addition, the hexamer dissociates into inactive dimers as the changes affect the loops at the hexamer interfaces. Extensive Dynamic Light Scattering and gel filtration studies showed that these transitions are triggered by pH changes.

## Conclusion

As a extension of the analysis of the hot dog fold present in the structure of the enzyme FabZ of *P. falciparum *that we determined [6], we probed the nature of this fold present in other proteins as well. Our analysis led to the identification of the probable functions of the hypothetical proteins containing the hot dog fold through detailed structural comparisons and identification of sequence motifs. Our analysis also demonstrates that quaternary association and function are related in certain cases. The consensus sequence motifs localized to the interface of quaternary structure and active site direct the mode of association and substrate specificity. Different modes of quaternary association were observed in the MaoC subfamily where the consensus sequence motif at the interface varies considerably. Double hot dog domains might have evolved by gene duplication to accommodate bulky substrates and to regulate activity by the asymmetric induced fit mechanism. It appears to be a case of directed divergent evolution where sequences changed substantially to design various types of quaternary association to carry out different functions with the same fold.

## Methods

### Functional annotation of hot dog fold proteins

All non-redundant crystal structures containing the hot dog fold in the PDB were identified using the DALI server hosted by the EMBL-EBI [45] using a dimer of FabZ from *P. falciparum *(PDB code: 1Z6B) as a search model. The sequence of one representative structure from each subfamily obtained from the DALI search was submitted to the Blastp server of NCBI [46] and a search for homologues in the PDB was carried out which resulted in the identification of all the structures with the hot dog fold within the subfamilies. A total of 64 non-redundant crystal structures were considered for the present analysis. Out of these structures, more than forty are from various structural genomics programs and have not been biochemically characterized so far. We attempted to assign functions to these proteins based on sequence and structural analysis. These crystal structures were grouped into respective subfamilies according to the presence of consensus sequence motifs [2] and available biochemical information. For the structures where the consensus sequences were not strictly followed, homologous proteins were picked up from the Swissprot database using the BLAST server. The consensus sequence motifs could be readily identified in these homologous proteins and these were used to assign the function. The crystal structures were visually examined in PyMOL [DeLano Scientific LLC] to identify the repeating structural unit. The possible quaternary association was identified by displaying the symmetry-related molecules and was further confirmed by examining the buried surface areas using the server MSDpisa [47]. For the hypothetical proteins classified into structurally and functionally characterized subfamilies, the functional annotation was confirmed by the similarities in the mode of quaternary association and structural conservation of the active site architecture. Pairwise structural superposition of the monomers and dimers was carried out using DaliLite. These results are used to calculate the structural distance measure (SDM) [48,49]. SDM values are calculated based on structural superposition and thus provide a better model for the evolutionary relation of proteins with very low sequence identities. MEGA [44] is used to generate the final phylogenetic tree using the distance matrix of the SDM values.

## Authors' contributions

Theme of the paper originated from KS and LSP and the paper was written by them. LSP, KR and KM carried out the work and analyzed the data. KS and NS were involved in the final interpretation of the results. All authors read and approved the paper.

## Supplementary Material

Additional file 1 **Proteins containing the hot dog fold in the PDB**. Protein Data Bank files of all the proteins used in this analysis along with their organism names and oligomeric states. Click here for file

Additional file 2 **Consensus sequence motifs**. Signature consensus motifs of each subfamily of the hot dog fold proteins and the corresponding sequences present in the proteins used in the present analysis. Click here for file
