# Supplementary material for: Analysis of proteins with the 'hot dog' fold: Prediction of function and identification of catalytic residues of hypothetical proteins
Source: BMC Struct Biol. 2009 May 28;9:37. doi: 10.1186/1472-6807-9-37 (PMC2698920; doi:10.1186/1472-6807-9-37)
Supplement: Additional file 1 — Proteins containing the hot dog fold in the PDB. Protein Data Bank files of all the proteins used in this analysis along with their organism names and oligomeric states. [file 1472-6807-9-37-S1.doc]

| **Subfamily** | **PDB code** | **Biological assembly** |
| --- | --- | --- |
| **1.Dehydratases**  **a) FabA**  *E. coli* | 1MKB | D |
| **b) FabZ**  *P. aeruginosa*  *P. falciparum*  *P. falciparum*    *H. pylori* | 1U1Z 1Z6B  1ZHG  2GLL | H1  H1  D  (low pH)  H1 |
| **2. Thioesterases**  **a. Acyl-CoA Thioesterases**  *B. halodurans*  *H. influenzae*  *B. cereus*  *A. tumefaciens*  Human  Mouse N-terminal domain  C-terminal domain  *T. thermophilus*  Human | **1VPM**  1YLI  **1Y7U**  **2GVH**  **2QQ2**  2V1O  2Q2B  **2EIS**  **3B7K** | H2  H2  H2  Tr_dh_  H2  H2  D  H2  Tr_dh_ |
| **b. TesB –like Thioesterases**  *E. coli*  Yeast | 1C8U  **1TBU** | D_dh_B  TB |
| **c. YbgC-like Thioesterases**  *E. coli*  *T. thermophilus*  *S. solfataricus*  *P. marinus*  *A. aeolicus* | **1S5U**  **1Z54**  **2GF6**  **2HX5**  **2EGJ** | TA  TA  TA  TA  TA |
| **d. Hydroxyacyl-CoA dehydrogenase-associated thioesterases**  *P. putida* | **2HLJ** | D |
| **e. 4-hydroxybenzoyl-CoA thioesterases** (degradation of 4-chlorobenzoate)  **i) 4HBT-I** *Pseudomonas sp. CBS-3*    *Jannaschia sp. CCS1* | 1BVQ &  1LO7  **2OAF** | TA  TA |
| **ii) 4HBT-II**  *Arthobacter sp. Strain SU*  *H. influenzae*  *E. coli*  *E. coli* | 1Q4T  **1O0I**  **1VH5**  **1VH9** | TB  TB*  TB*  TB* |
| **f. Paa I**  *T. thermophilus*  *E. coli*  *P. aeruginosa*  *M. musculus*  Human  *Silicibacter sp.*  *R. eutropha*  *T. fusca*  *P. horikoshii*  *C. crescentus*  *S. loihica*  *Rhodococcus sp.* | 1J1Y  2FS2  **1ZKI**  **2CY9**  2F0X  **2QWZ**  **2PIM**  **3BBJ**  **1IXL**  **2HBO**  **2PRX**  **2OV9** | TB  TB  TB  TB  TB  TB  TB  D_dh_B  D  D  D  D |
| **g. Fat** (Acyl-ACP thioesterase localized to plastids)  *B. thetaiotaomicron*  *L. plantarum* | **2ESS**  **2OWN** | D_dh_A  D_dh_A |
| **3. R-specific enoyl-CoA hydratases**  **a. MaoC-like hydratases**  *A. caviae*  *C. tropicalis*  Human peroxisomal  *M. tuberculosis*   1. *fulgidus*   *A. fulgidus*  Yeast | 1IQ6  1PN2  1S9C  2BI0  **1Q6W 2B3M**  2UV8 | D  D_dh_A  D_dh_A  Tr_dh_  H2  D  T^+^ |
| **b. NodN-like** *M. tuberculosis* | 2C2I | D |
| **4. YbaW**  *E. coli*  *P. aeruginosa*  *P. aeruginosa*  *T. thermophilus*  *X. campestris*  *Jannaschia sp. CCS1*  *B. stearothermophilus* | **1NJK**  **2ALI**  **2AV9**  **2CYE**  2FUJ **2NUJ**  **2OIW** | TA  TA*  TA  TA  TA  TA  TA |
| **5. FapR**  *B. subtilis* | 2F3X | D |
| **6. Acetyl transferase**  *S. oneidensis*  *P. aeruginosa*  *P. aeruginosa* | **1T82**  **1SH8**  **1YOC** | D  D  D |
| **New proteins**  *T. thermophilus*  *T. maritima*  *M. tuberculosis* | **2CWZ**  **2Q78**  2PFC | D  D  H3 |

* Oligomers identified by the present analysis.

^+^ Triple hot dog fold containing a double hot dog like fold between the first and the third domain.

Hypothetical proteins are shown in bold.

Proteins for which subfamilies are assigned by the present analysis are underlined.

These were either not annotated or differently annotated in the PDB files.
