# Supplementary material for: Analysis of proteins with the 'hot dog' fold: Prediction of function and identification of catalytic residues of hypothetical proteins
Source: BMC Struct Biol. 2009 May 28;9:37. doi: 10.1186/1472-6807-9-37 (PMC2698920; doi:10.1186/1472-6807-9-37)
Supplement: Additional file 2 — Consensus sequence motifs. Signature consensus motifs of each subfamily of the hot dog fold proteins and the corresponding sequences present in the proteins used in the present analysis. [file 1472-6807-9-37-S2.doc]

| **PDB** | **Consensus sequence motifs** |
| --- | --- |
| **1.Dehydratases**  **a) FabA**  (Dehydratase/ Isomerase involved in typeII FAS catalyzing the dehydration of β-hydroxyacyl-ACP to *trans-*2-enoyl-ACP and isomerization of *trans-*2-enoyl-ACP to *cis-*3-acyl-ACP)  1MKB | 1) MLM-x-DRI  2) AELDI-x-PDLWFF-x-C**H**F-x-DPVMPGCLGL**D**AMWQL-x-GF-x-LGW-x-G  3) RALG-x-GEVKF-x-GQVLP  1) MLMMDRV  2) AELDINPDLWFFGC**H**FIGDPVMPGCLGL**D**AMWQLVGFYLGWLG  3) RALGVGEVKFTGQVLP |
| **b) FabZ**  (Dehydratase involved in typeII FAS catalyzing the dehydration of β-hydroxyacyl-ACP to *trans-*2-enoyl-ACP)  1U1Z  1Z6B & 1ZHG    2GLL | 1) HRYPFLLVD  2) KNVT-x-NE  3) **H**FPP-x-MPGVLI-x-**E**AMAQ  1) HRYPFLLVD  2) KNVSINE  3) **H**FPEHPIMPGVLII**E**AMAQ  1) HRYPFLLVD  2) KQVSTNE  3) **H**FPQKQIMPGVLQI**E**ALAQ  1) HRYPMLLVD  2) KNITFNE  3) **H**FPNKPIFPGVLIV**E**GMAQ |
| **2. Thioesterases**  **a. Acyl-CoAs Thioesterases** (hydrolysis of acyl-CoA thioesters to free fatty acids and CoA)  **1VPM**  **1YLI**  **1Y7U**  **2GVH**    2V1O  2QQ2  **2Q2B**  **2EIS**  **3B7K** | 1) FGG-x(2)-M-x(2)-D  1) FGGKVLAYI**D**  1) FGGWIMSQM**D**  1) FGGKILSEM**D**  1) FGGTGLALM**D**  FGGEAIAYMT  1) HGGTILKMIE  1) HGGVTMKLM**D**  1) HGGVTMKLM**D**  1) FGGTVLAWM**D**  1) SAGQLLKWI**D**  FGGQIMAWME |
| **b. TesB –like Thioesterases**  (hydrolysis of acyl-CoA thioesters to free fatty acids and CoA in peroxisomes)    1C8U  **1TBU** | 1) VFGGQ  2) GQAL-x-AA  3) VHSLH-x-YFLR-x-GD  4) VER-x-RDG-x-SFS-RRV-x-A-x-Q-x-G  5) LAY-x-S**D**  6) A**S**LDHSMWFH  7) **Q**EG-x(2)-R   | 1) VFGGQ  2) GQALYAA  3) VHSFHSYFLRPGD  4) VETLRDGNSFSARRVAAIQNG  5) LGYAS**D**  6) A**T**IDHSMWFH  7) **Q**EGVMR  1) TFGGT  2) SQSLLAS  3) PTSLHSYFIKGGD  4) VQNLRNGRNFIHKQVSAYQHD | | --- | |
| **c. YbgC-like thioesterases**  (hydrolysis of short chain aliphatic acyl-CoAs in *tol-pal* cluster)  **1S5U**  **1Z54**  **2GF6**  **2HX5**  **2EGJ** | 1) DT**D**-x(2)-GVV-x-H-x(2)-Y  1) DT**D**AGGVVYHASY  1) ET**D**QMGVVHHSVY  1) DT**D**AQGIAHYAAY  1) DT**D**AAGGVMHFHQLF  1) ET**D**AQGIVHHSNY |
| **d. 3-Hydroxyacyl-CoA dehydrogenase-associated thioesterases**  (catalyzes the reduction of 3-hydroxyacyl-CoA to 3-oxoacyl-CoA in fatty acid metabolism)  **2HLJ** | 1) WIDYNGHM-x-DA-x-Y  1) WVDYNGHLRDAFY |
| **e. 4-hydroxybenzoyl-CoA thioesterases** (degradation of 4-chloro benzoate catalyzing the conversion of 4-chlorophenacyl-CoA to 4-hydroxybenzyl-CoA)  **(i) 4HBT-I**    1BVQ & 1LO7    **2OAF** | 1) FGDC**D**PAGIVWFPNYFRWLDAASRHFFI-x-CG  2) SYDDVL-x-IET-x-IREW-x-RKSF-x-Q-x-H-x-V  3) QLVM-x-ADETRVFAG  4) GRLRAIPIPADY  1) FGDC**D**PAGIVWYPNYHRWLDAASRNYFIKCG  2) SYDDVLTIETCIKEWRRKSFVQRHSV  3) QLVMRADEIRVFAM  4) GERLRAIEVPADY    1) WGDC**D**PAKIAYTGHLPRFALEAIDAWWSEYHG  2) TPRHILKCHTWPTRLGTKSITFRVDGV  3) GVTCFVGAFTCVFTI  4) QFKSQPAPDHLRA |
| **(ii) 4HBT –II**    1Q4T  **1O0I**  **1VH5**  **1VH9** | 1) MPVD-x-RT-x-QPFG-x-LHGGAS-x-LA**E**  2) VGLE-x-NANH-x-RS  3) TQVW-x-I-x-I-x-DE  1) VEVTDLRQRWGLVHGGAYCALA**E**  2) VGQSNHTSFF  3) TTWFWDVSLRDD  1) MPVDHRTMQPFGVLHGGVSVALA**E**  2) VGLDINANHLRPVRS  3) IQVWQIDIRTEE    1) MPVDSRTKQPFGLLHGGASVVLA**E**  2) VGLEINANHVRS  3) HQVWQIEIFDE  1) MPVDTRTHQPFGLLHGGASAALA**E**  2) VGTELNATHHRPVS  3) NQSWEIVVFDE |
| **f. Paa I thioesterases**  ( Phenyl acetic acid degradation)    1J1Y  2FS2  **1ZKI**  **2CY9**  2F0X  **2QWZ**  **2PIM**  **3BBJ**  **1IXL**  **2HBO**      **2PRX**    **2OV9** | 1) HGG-x-IF-x-LA**D**  2) GRTG-x-YDV-x-V-N-x(2)-G-x-LVA-x-FRG-x-SYR  1) HGGFLYALA**D**  2) SRRTATYRVEVVSEGKLVALFTGTVFR  1) HGGQLFSLA**D**  2) GKQTGVYDIEIVNQQQKTVALFRGKSHR    1) HGGALFSLM**D**  2) GRRSLVVEAEVRQGDKLVAKGQGTFAQ  1) HGGLTATLV**D**  2) GKTLAFASVDLTNKTTGKLIAQGRHTKHL  1 )HGGLTATLV**D**  2) GKTLAFTSVDLTNKATGKLIAQGRHTKHL  1) SGPSMFALA**D**  2) GRTLAVGDILLFSEGMEAPVARSTMTYSI  1) QGGMLGAML**D**  2) GRNVCNVVGELSQDGKLVATATATCMV  1) NGGYLMTVLQ  LAFLPLAV**D**  2) GRTVTTVQTTLFQEGRTILTGTLATATL  SDGWFDENVDLWDARGRLVAQSRQLARV  1) HGGFTFGLA**D**  2) GKKKIVEVKVYREEEVVLEGKFYCYV  1) HGGMLMSFA**D**  2) EDMLFTVRGRIWAGERTLITGTGVFKA  1) YGGLIASLI**D**  2) RKVVVEIALSALCARGHMVAVK  1) HGGVSALLL**D**  2) DGRKITTAGDIRTADGQVCVSVEGLFVD |
| **g. Fat thioesterases specific to acyl-ACPs**  (Acyl-ACP thioesterase localized to plastids catalyzing the termination of type II fatty acid biosynthesis via cleavage of acyl chain from ACP)  **2ESS**  **2OWN** | 1) LIWVV-x(2)-M-x-I  2) DLD-x-NQHVN NVKYIGW-x-LES  1) YTWVLSRLAI  2) DIDINGHVNSIRYIEHILDL    1) VGWVV TQYAI  2) DIDPNRHVNNAHYFDWLVDT |
| **3. R-specific enoyl-CoA hydratases**  **a. MaoC-like**  (hydrate trans-2-enoyl-CoA to (R)-3-hydroxyacyl-CoA in polyhydroxy-alkanoate (PHA) biosynthesis pathway)  1IQ6  1PN2  1S9C  2BI0  **1Q6W**  **2B3M**  2UV8  **b. NodN-like**  (Nodule formation in plants)  **2C2I** | 1) [YF]-x(1,2)-[LIVG]-[STGC]-G**D**xNP-[LIV]-**H**x(5)-[AS] **+**  1) FAALSE**D**FNPL**H**LDPAFAA  1) YNIALGATEKQLKYVYENDS  YRLSG**D**RNPL**H**IDPNFAK    1) YALGVGASIKDPKDLKFIYEGSS  YRLSG**D**WNPL**H**IDPNFAS  1) HQSIVGNRLRLALDSDLCA  LARLTL**N**IAAT**H**HDWRVS  1) FAYLTA**D**FFPL**H**TDVEFAK  1) FGLISG**D**LNPV**H**FDEDFAS  1) YARVSG**D**LNPI**H**VSRHFAS  1) FADAT-x-**D**HQWI**H**VDPERAA  2) PFG-x-TIAHGFLTLSLLP  3) NYG-x-DKVRF-x(2)-PV-x(2)-GSRVR  4) TVEIEG-x(2)-KPA-x-VAE  1) FADATG**D**HQWI**H**VDPERAA  2) PFGTTIAHGFMTLALLP  3) NYGLNKVRFPAPVPVG  4) TVEVEGSAKPACVAE |
| **4. YbaW (conserved hypothetical Protein)**    **1NJK**  **2ALI**  **2AV9**  **2CYE**  2FUJ  **2NUJ**  **2OIW** | 1) IKVRGYHL**D**VYQHVNNARYLEFLEEARW  2) VVNININYRRPAVLGDLL  3) ALITFVCIDLKTQKAL-x-LEGELREKLE  1) IKVRGYHL**D**VYQHVNNARYLEFLEEAARW  2) VVNININYRRPAVLSDLL  3) ALITFVCIDLKTQKALALEGELREKLE    1) IPVRWGDM**D**SYGHVNNTLYFQYLEEARV  2) VVLQSLHTYLKPVVHPATV  3) GHCKLVWVRHAENRSTPVP  1) ISTRWHDN**D**IYGHVNNVTYYAFFDTAVN  2) LVVSSSCDYFAPVAFPQRI  3) GRFVHVFVERRSSRPVAIP  1) VDVRFRDL**D**PLGHVNNAVFLSYMELARI  2) VVARMEVDYLRPILLGDEV  3) GLGVLVWLEGGRPAPLP  1) ISVRWRDM**D**SMGHVNNAKYISYLEEARV  2) VVAATNVNYKRPLVWPNDI  3) GNVVVVWIDTQTGKSASLP  1) DRVRFGEL**D**AIGHVNHTAYLRWYESFRL  2) VLKQVHCTYLAEMGMGEDY  3) GSAVVVLLNRDGSGRYPIP  1) ITPRVSET**D**GVGHINNTTVPVWFEAGRH  2) VIIRMEVDYVNQMYYGQDV  3) GRSVYVNFNFDTGRPEPI |
| **5. FapR**  (Transcriptional regulation of fatty acid metabolism)  2F3X | 1) IARGHHLFAQANSLAVAVI-x(2)-E-x-ALT  2) GERV-x-AKA-x-V  1) IARGHHLFAQANSLAVAVIDDELALT  2) GERVVAKAKV |
| **6. Acetyl transferase**  (Protein YiiD with a fused acetyl transferase domain)  **1T82***  **1SH8***  **1YOC*** | 1) SEKMGV-x-I-x-QYTGQ-x-F  2) APL-x-PN-x-NPH-x-T-x-FAGS  3a) LATLTGWGL-x-WL-x-L-x-ER-x-L-GDIVLAD-x(2)-IRY  3b) SGDLDRLA-x-GRKAR  4) GTY-x-VLP  1) SEFMQIAPLSFTDGELS  2) APLAPNINLHHTMFAGS  3a) IM**T**LTGWGMVWLQQQLLNVDGDIVLADAHIRY  4) DTNSPLQ    1) QRSGLRAEVLEPGYV  2) MPGAGNENHIGSMYAGA  3a) LA**E**LPGGALFLTSFDSARFYPIVKEMTLRFRR  1) FASIAPQFVELRPGYAE  2) FPKRREVLNHIGTVHAI  3a) AA**E**LAAGTMTDASIPAGHRWIPRGMTVEY |

Consensus sequences are given in blue and the actual sequences in black.

Sequences shown in green are less similar to the Consensus sequence.

^+^The consensus sequence motif identified by Qin *et al*. [Qin YM, Haapalainen AM, Kilpelainen SH, Marttila MS, Koski MK, Glumoff T, Novikov DK, Hiltunen JK: **Human peroxisomal multifunctional enzyme type 2. Site-directed mutagenesis studies show the importance of two protic residues for 2-enoyl-CoA hydratase 2 activity**. *J Biol Chem* 2000, **275**(7):4965-4972] while the others by Dillon & Bateman [Dillon SC, Bateman A: **The Hotdog fold: wrapping up a superfamily of thioesterases and dehydratases**. *BMC Bioinformatics* 2004, **5**:109].

* The proteins whose consensus sequence motif was first identified in the homologous proteins that are annotated.

The catalytic residues are shown in bold.
